# First field evidence of aerosolised SGPV, ISAV-HPR0, and IPNV in Atlantic salmon RAS highlights transmission and biosecurity risks

**DOI:** 10.1038/s41598-025-21970-y

**Published:** 2025-10-30

**Authors:** Dhiraj Krishna, Petra Elisabeth Petersen, Maria Marjunardóttir Dahl, Ingibjørg Egholm, Debes Hammershaimb Christiansen

**Affiliations:** 1 National Reference Laboratory for Fish and Animal Diseases, Faroese Food and Veterinary Authority, Torshavn, Faroe Islands; 2 Department of Veterinary and Animal Sciences, University of Copenhagen, Copenhagen, Denmark; 3 Hiddenfjord, Sandavágur, Faroe Islands

**Keywords:** Aerosolised eDNA/eRNA, Anaesthetic water, Atlantic salmon pathogen surveillance, Coriolis Compact, Coriolis+, RAS biosecurity, Diseases, Environmental sciences, Microbiology

## Abstract

Recirculating aquaculture systems (RAS) for Atlantic salmon (*Salmo salar* L.) are closed-containment systems (CCS) offering biosecure, water-efficient farming conditions, yet pathogen transmission remains a critical concern. While horizontal transmission in water is well-documented in RAS, the potential for aerosol-mediated transmission remains underexplored. The current study was conducted at two commercial Faroese Atlantic salmon RAS smolt farms. At Smolt farm 1, aerosolised pathogens were monitored using two aerosol samplers (Coriolis+ and Coriolis Compact, Bertin Technologies SAS, France), along with water and fish swab samples, to evaluate pathogen dynamics. A sequential infection pattern was observed, beginning with salmon gill pox virus (SGPV), followed by non-virulent infectious salmon anaemia virus (ISAV-HPR0), piscine orthoreovirus-1 (PRV-1), and sporadic detections of infectious pancreatic necrosis virus (IPNV) and *Flavobacterium psychrophilum*. All pathogens were detected in aerosol samples, with the highest detection rates and pathogen loads at the biofilter room compared to the local tank degassers. Detection trends for SGPV and ISAV-HPR0 in aerosols reflected those in fish and water samples. Coriolis+ performed marginally better compared to Coriolis Compact in reflecting the infection dynamics. Viable IPNV was not isolated from initial aerosol samples at Smolt farm 1, though bacterial culture identified relevant colonies for Atlantic salmon RAS. Targeted aerosol sampling for IPNV at Smolt farm 2 post IPNV outbreak produced IPNV-specific cytopathic effects in cell lines from Coriolis Compact aerosol samples, marking the first field-based evidence of viable aerosolised IPNV from a RAS. The current study extends our previous work by introducing anaesthetic water as a refined, non-invasive surveillance method, whilst providing the first field-based evidence of Atlantic salmon viruses in RAS aerosols, which signals the potential for airborne transmission and emphasises the need for strict biosecurity measures.

## Introduction

Pathogens have long posed significant challenges in aquaculture, leading to considerable economic losses^1^. In recent years, advances in technical expertise have made Recirculating Aquaculture Systems (RAS) increasingly prominent and widely used in intensive fish farming^2^. Due to high water retention, potential pathogens can accumulate over time if water quality parameters are not optimally maintained, allowing them to establish in various RAS components, most notably in biofilms, which are known to harbour pathogens^3–5^. Outbreaks in RAS are linked to various pathogens, often triggered by environmental fluctuations^2^ and are largely dependent on the fish species being cultivated^4^. Atlantic salmon (*Salmo salar*), a highly valued species, is intensively farmed in several countries, with a significant portion of production attributed to Norway, Chile, Scotland, Canada, Iceland and the Faroe Islands, which use intensive systems like RAS for rearing the freshwater pre-smolt stage^6,7^. Like other cultured fish species, Atlantic salmon is significantly affected by various pathogens. Key pathogens relevant to the freshwater smolt production stage in RAS in the Faroe Islands include non-virulent infectious salmon anaemia virus (ISAV-HPR0), infectious pancreatic necrosis virus (IPNV), piscine orthoreovirus (PRV-1), salmon gill pox virus (SGPV) and *Flavobacterium psychrophilum*, along with opportunistic fungal pathogens like *Saprolegnia parasitica*^8^ (Faroese Food and Veterinary Authority; FFVA, *unpublished data*).

In aquaculture systems, fish pathogens can be transmitted horizontally from fish to fish, through fomites in water or feed^4^, and vertically from broodstock to progeny^9^. However, the exact transmission pathways are still unclear due to the diversity of fish species and their associated pathogens^10,11^. As most so-called vertical transmission results from contaminated egg surfaces rather than true vertical mechanisms (transovarial transmission)^10^, effective disinfection protocols are essential to reduce pathogen risk^12^. Of the above-mentioned pathogens, true vertical transmission (with preliminary evidence for Atlantic salmon) has been documented for IPNV^13,14^ and *Flavobacterium psychrophilum*^9^. Aerosol transmission of respiratory pathogens is well-documented in humans and terrestrial animals^15,16^, but whether fish pathogens, especially viruses, can also spread horizontally via aerosols remains unclear. Research on aerosol-mediated transmission of fish viral pathogens is scarce, though experimental evidence exists for non-viral pathogens like *Ichthyophthirius multifiliis*,* Amyloodinium ocellatum*,* Aeromonas hydrophila*, *A. salmonicida* and *Vibrio parahaemolyticus*^17–21^. Studies have shown that marine aerosols, produced by the bubbling of the sea surface microlayer (SML), are enriched with viruses and bacteria, enabling the dispersal of potential pathogens over long distances^22,23^. This phenomenon is also evident in sea spray, which can carry human pathogens and further extend microbial transport^24,25^. The spread of aerosols generated by the SML could explain the presence of genetically identical microbes in different geographical areas with no apparent connection^22^. RAS, as an intensive system, includes components that can generate aerosols from water. For example, the degassers, which draw in large amounts of air to remove carbon dioxide (CO_2_) from the water, and the drum filters, which generate aerosols directly through the mechanical action of spraying water^26,27^, are similar in characteristics to sea spray formation in the marine environment. When a RAS system experiences a pathogen outbreak, these pathogens may be continuously aerosolised and transmitted^20^.

We previously demonstrated clear associations between fish and water samples for the pathogens ISAV-HPR0 and SGPV in RAS^28^. In the current study, we expand the findings by employing two sampling strategies across two commercial Atlantic salmon smolt RAS farms. Over nine weeks, we monitored five key Faroese RAS pathogens: ISAV-HPR0, IPNV, PRV-1, SGPV, and *F. psychrophilum* in fish, water, and aerosol samples from Smolt farm 1. IPNV targeted aerosol sampling was conducted at Smolt farm 2, as IPNV is the only viral pathogen in the current study that can be propagated in cell lines^29^, making it the ideal “proxy” for testing the presence of viable virus in aerosol samples. Attempts were also made to isolate *F. psychrophilum* in pure culture from aerosol samples from Smolt farm 1. In addition to validating anaesthetic water sampling as an enhanced non-invasive early detection method and elucidating Atlantic salmon pathogen dynamics in multiple sample matrices, we present the first field evidence of aerosolised viable IPNV in RAS, underscoring the potential role of aerosols in pathogen transmission and the need for robust biosecurity in Atlantic salmon RAS.

## Materials and methods

### Ethics declarations

The current study was conducted per local legislation and regulations after obtaining ethics approval from the Faroese Food and Veterinary Authority (FFVA) (https://www.hfs.fo/). The study was conducted at the two smolt farms (Smolt farm 1 and 2) after obtaining all necessary permissions from the smolt farm authorities as well as the FFVA. All experimental protocols, fish handling, and sampling procedures were approved and supervised by the veterinarians at the smolt farms. The study adhered to the ARRIVE guidelines (https://arriveguidelines.org) for reporting of in vivo animal experiments.

### Longitudinal study comparing pathogen loads in aerosols with prevalence in fish swabs and water samples

**Fig. 1:**
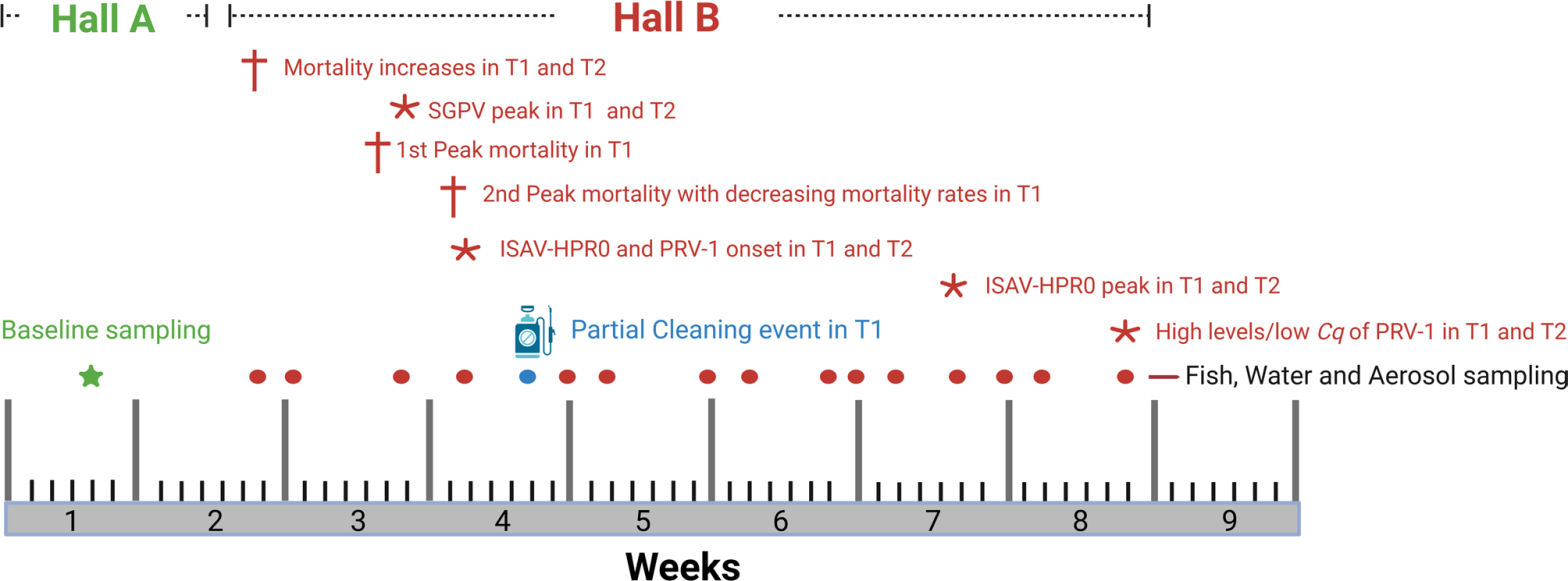
Sampling timeline highlighting critical events in the RAS at Smolt farm 1. The two halls represent two independently functioning RAS units. The fifth sampling point in hall B coincides with a partial cleaning event of T1 (*Figure created with BioRender*).

Sampling was conducted at Smolt farm 1, a full-scale commercial freshwater Atlantic salmon smolt RAS facility, an indoor closed-containment system (CCS), to investigate pathogen infection dynamics and evaluate potential pathogen load in aerosol samples. The study involved two RAS systems, hall A and hall B. Hall A contained 12 tanks, each with a capacity of 50 m^3^, holding naïve fish, free from the pathogens of interest and with no previous outbreaks, from which baseline samples were collected one week before the fish were moved to hall B (moved once the fish attained size range of 20–50 g). In hall B, sampling was performed at 16 time points (due to RAS maintenance, aerosol sampling was not conducted at the final point; thus, 15 time points for aerosol sampling) (Fig. 1). Hall B comprised eight tanks sharing a common biofilter. Two of the tanks (T1 and T2), each with a capacity of 320 m^3^, were included in the study. The remaining tanks housed non-naïve fish from a previous fish group, which were examined in our earlier work^28^ and were excluded from the current study. At the fifth sampling point:14 days post-introduction (dpi) of the last fish into hall B, a partial cleaning of T1 involving diluting tank water with fresh intake water was performed. This event, along with other key events, is highlighted in Fig. 1, while Table 1 details the total number of samples collected.

#### Collection of gill and kidney swabs

All samples were collected from pre-smolts before vaccination. Random sampling was performed for baseline samples collected from all 12 hall A tanks (Table 1). After anaesthetising the fish in buffered (NaHCO_3_; sodium bicarbonate; Apoteksverkið, Faroe Islands) Finquel Vet (MS 222, Merck & Co. Inc., USA) using 5 L of individual tank water, non-lethal gill swabs were collected directly into 600 µL of RLT lysis buffer (Qiagen, GmbH). Post fish transfer from hall A to hall B, seven pre-smolts per tank were collected at each sampling point, primarily consisting of freshly dead and moribund fish/fish displaying abnormal behaviour (lethargy, erratic swimming, abnormal breathing, loss of response to stimuli). Fish were euthanised with an overdose of benzocaine (Apoteksverkið, Faroe Islands) and gill and kidney swabs collected in 600 µL of RLT lysis buffer (Qiagen, GmbH) using sterile cotton swabs (Heinz Herez Hamburg, Germany). Gill swabs were used to test for SGPV, ISAV, and *F. psychrophilum*, while kidney swabs were used to test for IPNV and PRV-1. Uninoculated RLT lysis buffer (Qiagen, GmbH) was used as a field sample control. Sample collection followed the method described in Krishna et al.^28^. All samples were collected in the presence of a registered in-house veterinarian, adhering to guidelines set by local authorities. The total number of samples is indicated in Table 1.

#### Collection and filtration of water samples

Disposable 1-litre polyethene bottles (Identipack BV, Netherlands) were used to collect water samples. The bottles were submerged approximately 10 cm below the water surface. 1-litre water samples were collected from both the primary (main culture tank; PT) and anaesthetic tanks (AT) in halls A and B, corresponding to the fish samples. The total number of water samples collected in Smolt farm 1 is shown in Table 1. Upon receipt in the laboratory, the water samples were filtered using a 0.45 μm mixed ester membrane filter (Advantec MFS, USA) on a WaterVac 100 Filtration System (Rocker Scientific Co., Taiwan) until saturation^28^. The filters were then placed in 1 mL RLT lysis buffer (Qiagen, GmbH) and stored at − 20 °C until further processing. Blank RLT lysis buffer (Qiagen, GmbH) were used as a control.

**Table 1:** Total number of samples collected at their respective collection locations for smolt farm 1.

	Hall A (Baseline)					Hall B											
Sample type	Fish	Water		Aerosol		Fish		Water				Aerosol					
	Swab	Tank water	Anaesthetic water	Coriolis Compact	Coriolis+	Swab		Tank water		Anaesthetic water		Coriolis Compact			Coriolis+		
Location	T1–12	T1–12		Centre of the hall and biofilter		T1	T2	T1	T2	T1	T2	T1 Degasser	T2 Degasser	Biofilter	T1 Degasser	T2 Degasser	Biofilter
Number	*N* = 168 (*n* = 14)	*N* = 12	*N* = 12	*N* = 3	*N* = 3	*N* = 112 (*n* = 7)	*N* = 112 (*n* = 7)	*N* = 16	*N* = 16	*N* = 16	*N* = 16	*N* = 15	*N* = 15	*N* = 15	*N* = 15	*N* = 15	*N* = 15

‘*N*’ denotes the total number of samples, while ‘*n*’ indicates the number of samples gathered per sampling point per tank. ‘*T*’ refers to tanks. In hall A, aerosol samples were collected at the centre of the hall and the biofilter room (covering both the drum filter and the degasser aerosols), while in hall B, samples were collected at the local tank degassers and the biofilter room (as in hall A).

#### Collection and processing of aerosol samples

Two commercially validated aerosol samplers, the Coriolis+ wet cyclonic and the Coriolis Compact dry cyclonic (Bertin Technologies SAS, France), were used for aerosol collection. The Coriolis+ was operated at an airflow rate of 300 L/min for 10 min, while the Coriolis Compact operated at 50 L/min for 1 h, resulting in a total aerosol volume of 3000 L collected by each sampler. Samples were collected in UV-sterilised sampling cones (Saveen & Werner Aps, Sweden; Bertin Technologies SAS, France). For the Coriolis+, aerosol samples were collected in collection cones containing surfactant-based liquid (Bertin Technologies SAS, France). Sterile 1x Dulbecco’s phosphate-buffered saline without Ca^2+^ and Mg^2+^ (DPBS) (Corning, USA), containing 0.1% Ecosurf EH-9 (Triton X-100 replacement; Dow Inc., USA), was used as the collection liquid. In contrast, the Coriolis Compact relied on the adsorption of aerosol particles onto the walls of the collection cone without a collection liquid (Bertin Technologies SAS, France). Post aerosol sample collection, the cones from the Coriolis Compact were reconstituted with up to 2 mL of collection liquid, with immediate thorough mixing and vortexed upon receipt at the laboratory.

**Fig. 2:**
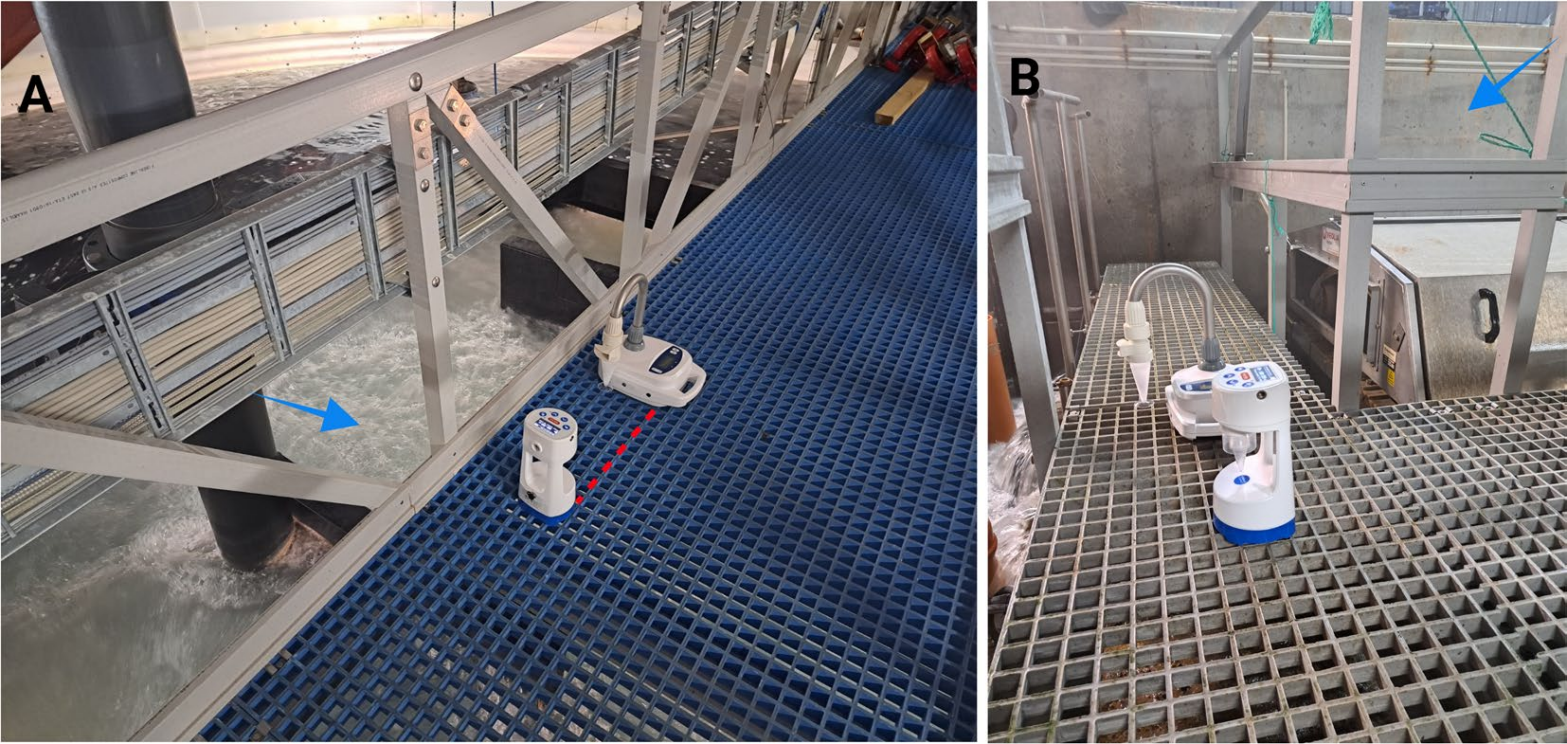
Aerosol sampling locations: tank local degassers (**A**) and the biofilter room (**B**). The two samplers were placed one foot apart in proximity to the source of aerosols. Arrows indicate positions of probable aerosol generation (*Figure created with BioRender*).

Baseline samples were collected in hall A, at the tank housing area (centre of hall), and the biofilter room (comprising a drum filter and degassers). After fish were transferred to hall B, sampling was conducted at three locations: the local degassers of the two tanks (Fig. 2A) and the biofilter room (comprising a drum filter and degassers) (Fig. 2B). The two samplers were placed ~ 1 foot apart, directly above the local degassers at the tank (Fig. 2A) and the centre of the biofilter room (Fig. 2B). Location selection and sampling durations were based on visual inspections and results from a pilot study (data not shown). Aerosol samples (corresponding to fish and water samples) were obtained at varying weekly frequencies: 15 sampling points in total, with three samplings per week around peak SGPV infection and peak mortality (Fig. 1; Table 1).

Amicon Ultra-15-50 kDa MWCO centrifugal concentrators (Merck & Co., Inc., USA)/ Vivaspin Turbo 4 30 kDa MWCO Ultrafiltration Unit (Sartorius AG, Germany) were used to concentrate the aerosol samples from the Coriolis+ (to increase the likelihood of pathogen detection). The walls of the centrifugal concentrators were pre-wetted with 2.5 ml of sterile 1x DPBS (Corning, USA) and spun at 2000×*g* for 2 min to minimise sample loss during centrifugation. The Coriolis+ aerosol samples were then transferred in parts and centrifuged at 2000×*g* for approximately 5 min or until the final volume in the upper chamber was reduced to about 500–1000 µL. All samples were stored at − 20 °C until further processing. Pre-concentration aliquots of 1 mL were preserved at − 70 °C for cell culture assays to assess the presence of viable viruses and bacteria. Duplicate unused sampling cones for each sampler were used to check for possible contamination and ensure sample integrity. A combination of ethanol and disinfectant wipes was used to decontaminate the samplers before and after sampling, involving running 70% ethanol-filled cones for 10 min and wiping the outer surface of the samplers as well as their attachments with Flow Wipes free (medichem Vertriebs GmbH) / PCR Clean Wipes (Minerva Biolabs GmbH).

#### Viability of aerosolised bacterial species and infectious pancreatic necrosis virus (IPNV)

Bacterial total plate count (TPC) and cell culture assay were used to assess the viability of *F. psychrophilum* and IPNV, respectively, in the aerosol samples. The spread plate method was employed to isolate bacterial colonies from the aerosol samples^30^. A sterile spreader was used to evenly plate 0.1 mL of aerosol samples from both samplers (including Coriolis+ concentrated samples) onto Cytophaga agar (CA) (selective for *Flavobacterium spp*). The plates were then incubated at 15 °C for 5–7 days, and bacterial colonies were manually counted. Individual colonies on the CA agar were subcultured onto fresh CA and Sheep Blood agar (BA) (Thermo Fisher Scientific, Inc., USA) to obtain pure cultures.

Cell lines were obtained from the National Institute of Aquatic Resources (DTU Aqua) at the Technical University of Denmark (DTU), Denmark. Two cell lines, Bluegill Fry (BF-2) and Epithelioma Papulosum Cyprini (EPC), were used in this study. BF-2 cells were cultured in Eagle’s Modified Essential Medium (EMEM) without Tris (Gibco, USA), supplemented with 10% sterile fetal calf serum (Gibco, USA), Penicillin-Streptomycin, and Amphotericin B (Gibco, USA) and incubated at room temperature (24 ± 2 °C) for 24 h until a uniform cell monolayer with at least 80% confluence was achieved^29^. The cells were washed three times with 1x DPBS (Gibco, USA), trypsinised with 1 mL TrypLE Express (Gibco, USA), and resuspended in new maintenance EMEM with Tris at a ratio of 1:3. A total of 1.5 ml of the cell suspension was transferred into 24-well polystyrene cell culture plates and incubated overnight at room temperature (24 ± 2 °C). Similarly, EPC cells were cultured in Leibovitz’s L-15 medium (Gibco, USA) containing Gentamicin (Gibco, USA) and 10% sterile fetal calf serum (Gibco, USA), following the same procedure as for BF-2 cells^29^. Individual wells of the cell culture plates were inoculated with 150 µL of the aerosol sample at three dilutions and incubated at 15 °C in a humid chamber. Uninoculated wells served as negative controls. The cells were checked daily for two weeks for the appearance of cytopathic effect (CPE). All cell culture wells were screened for IPNV using RT-qPCR, as described in Sect. “Quantitative reverse transcription polymerase chain reaction (RT-qPCR)”. When necessary, cell culture samples were passaged a second time after seven days of incubation.

#### Nucleic acid extraction

The preprocessing of fish swabs and membrane filter samples was done as previously described by Krishna et al.^28^. For aerosol samples, 200 µL of concentrated aerosol was mixed with 600 µL of RLT lysis buffer (Qiagen, GmbH) and incubated at room temperature for 15 min; 400 µL of this mixture was used for RNA extraction. The KingFisher Apex (Thermo Fisher Scientific Inc., USA) semi-automated nucleic acid extraction system was used according to the manufacturer’s instructions, together with the MagMAX Viral/Pathogen Nucleic Acid Isolation Kit (Applied Biosystems, USA) for fish, water, and aerosol samples (extracted in duplicate for aerosols). For cell culture samples, 200 µL of cell culture suspension was mixed with 600 µL of RLT lysis buffer (Qiagen, GmbH). RNA was extracted using the MagMAX Viral/Pathogen Ultra Nucleic Acid Isolation Kit (Applied Biosystems, USA) on the KingFisher Apex system. Individual bacterial colonies were transferred to ATL buffer (Qiagen, GmbH), homogenised, and DNA was extracted using the DNeasy Blood and Tissue Kit (Qiagen, GmbH) according to the manufacturer’s instructions. Eluted DNA/RNA was stored at − 20 °C. Uninoculated/blank RLT and ATL lysis buffer (Qiagen, GmbH) were used as extraction controls.

#### Quantitative reverse transcription polymerase chain reaction (RT-qPCR)

Duplex RT-qPCR was performed for ISAV, IPNV, PRV-1, SGPV and *F. psychrophilum* on the QuantStudio 5 (Applied Biosystems, USA) PCR system using sequence-specific TaqMan (Thermo Fisher Scientific Inc., USA) DNA hydrolysis probes as previously described^28^. The Elongation Factor 1 alpha (*EF1α*) gene of Atlantic salmon served as the housekeeping gene for swab and water samples, with synthetic DNA sequence/gBlocks (Integrated DNA Technologies Inc., USA) used as standards/positive controls and nuclease-free water as a no-template control (NTC) to validate the reactions. Additional screening of aerosol samples was performed using the 16S rRNA gene and internal transcribed spacer (ITS) of *Saprolegnia parasitica* as control genes to validate them. For the 16S rRNA gene, BactQuant primers^31^ were used with the QuantiTect probe PCR kit with the following temperature profile: 95 °C for 15 min, followed by 40 cycles of denaturation at 94 °C for 15 s and extension at 60 °C for 60 s. For the ITS region of *Saprolegnia parasitica*^32^, the TaqPath 1-step RT-qPCR kit was used under the following conditions: 50 °C for 15 min, followed by 95 °C for 2 min, and 40 cycles of denaturation at 95 °C for 3 s and extension at 60 °C for 30 s. Results were analysed using QuantStudio Design and Analysis Software v1.5.2 (Applied Biosystems, USA). All primers used in this study for the selected pathogens were in-house modifications from previous studies^5,33–38^, and sequences are available upon request.

#### Bacterial colony identification by next generation sequencing (NGS) of 16S rRNA gene

Identification of the bacterial colonies was based on sequencing the entire 16S rRNA gene (V1-V9; 1500 bp), performed using Long-Read Next Generation Sequencing (NGS). Universal 16S primers based on previous studies^39^ were used for initial amplification and sequencing. DNA samples were amplified in a 20 µL reaction mixture containing 10 µL of Q5 Hotstart High-Fidelity Master Mix (2X) (New England Biolabs, UK), 1 µL each of 10 µM 16S universal forward and reverse primers, 1 µL of colony-extracted DNA and molecular grade water to reach the final volume. PCR cycling conditions were as follows: initial denaturation at 98 °C for 30 s, followed by 35 cycles of 98 °C for 10 s, 62 °C for 20 s, and 72 °C for 1 min, using a SimpliAmp thermal cycler (Applied Biosystems, USA). Amplified DNA was quantified with a Qubit 4 fluorometer (Invitrogen, USA). All clean-up steps were performed using AMPure XP beads (Beckman Coulter Inc., USA) on a DynaMag-96 Side Magnet (Invitrogen, USA). Library preparation was done using the Native Barcoding kit (SQK-NBD114.24) as per the Ligation Sequencing Amplicon-Native Barcoding Kit 24 V14 protocol (Oxford Nanopore Technologies, UK). The final library (35–50 fmol concentration range) was loaded onto a primed R10.4.1 flow cell and sequenced on a GridION sequencer using super accurate base-calling (Oxford Nanopore Technologies, UK) until sufficient reads were obtained.

### Targeted sampling and culturing of aerosolised infectious pancreatic necrosis virus (IPNV)

Targeted sampling was carried out at Smolt farm 2, a freshwater commercial indoor CCS RAS, following an IPNV outbreak in QTL-IPN-resistant Atlantic salmon fry, reported during routine monitoring. Water and aerosol samples were collected at the biofilter room of the RAS system affected by the outbreak, with triplicate water and aerosol samples (for each aerosol sampler). A strict cold chain was maintained from sample collection through transportation, with expedited sample processing and inoculation into naïve cell monolayers. The water filtration, aerosol sample collection and processing, cell culture assay, RNA extraction, and RT-qPCR were conducted as previously described in Sect. “Collection and filtration of water samples” to “Quantitative Reverse Transcription Polymerase Chain Reaction (RT-qPCR)” for Smolt farm 1. The only exception to the aerosol sample collection protocol was using sterile DPBS (Corning, USA) without surfactant as the collection and reconstitution liquid for Coriolis+ and Coriolis Compact, respectively.

#### Genotyping infectious pancreatic necrosis virus (IPNV)

IPNV was genotyped based on the viral protein 2 gene (*VP2*). Samples collected during routine operations at Smolt farm 2 had previously been genotyped in-house as part of routine lab processing, and these data were made available for the present study. IPNV from cell culture samples were genotyped post-positive observation of CPE on cell lines and confirmation with IPNV RT-qPCR. Initial amplification was done with One RT-qPCR kit (Qiagen, GmbH) with purification and cycle sequencing as previously described^28^. The final products were sequenced on a SeqStudio Genetic Analyser (Applied Biosystems, USA). The IPNV primers used were an in-house modification based on previous studies^40,41^, and will be made available on request.

### Statistical and bioinformatics analyses

All statistical analyses and graphical illustrations were performed using GraphPad Prism v10.4.2 (GraphPad Software LLC, USA) and the BioRender web-based app (BioRender.com). Data normality was assessed with the D’Agostino-Pearson test, and correlations between sample types were evaluated using the Spearman non-parametric test (two-tailed). An *r*_*s*_ value close to 1 was considered a strong positive correlation, while values close to 0 were considered weak. The Mann-Whitney U test (two-tailed) was used to compare the two aerosol samplers. A *P*-value < 0.05 was considered significant in all analyses and indicated where appropriate. Sequence data from the SeqStudio were analysed with the Sanger Sequencing module in CLC Main Workbench (v24.0.2) (Qiagen, GmbH). Amino acid translations were obtained using the nucleotide translation module in the CLC Main Workbench (v24.0.2) (Qiagen, GmbH). For NGS data, base calling was performed with Dorado (v7.4.12) on MinKnow software (v24.06.10) (Oxford Nanopore Technologies, UK), incorporating built-in adapter removal and a minimum Q-score of 10. All reads passing (*Q-score > 10*) were further quality-filtered with Filtlong (v0.2.1)^42^ to retain high-quality reads between 600 and 1800 bp (*min_mean_q = 30*). The filtered reads were then assembled using the MEGAHIT de novo assembler (v1.2.9)^43^ and the *De novo* genome assembler module in CLC Genomic Workbench (v24.0.2) (Qiagen, GmbH) to identify distinct contigs. Primer sequences were manually trimmed from the contig sequences and subsequently checked for chimeric sequences using VSEARCH (v2.30.0)^44^. Nucleotide BLAST (megablast) was used to identify colonies based on the similarity of their final contigs.

## Results

### Aerosol samples validated using 16S rRNA and ITS of saprolegnia parasitica as control genes

**Fig. 3:**
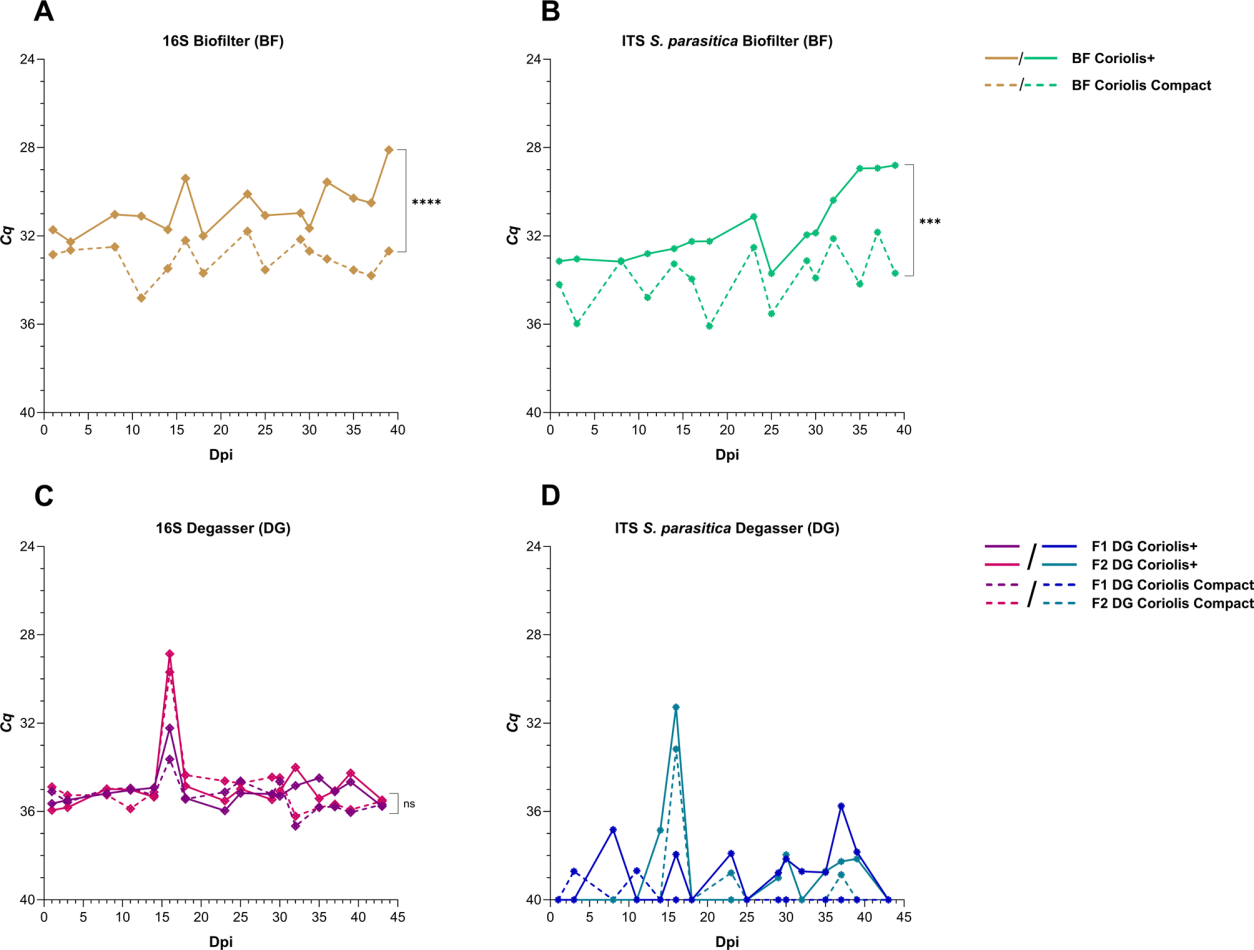
Detection levels of the control genes 16S rRNA and ITS of *S. parasitica* from biofilter room and local degasser aerosol samples. The y-axis displays the inverted mean cycle quantification (*Cq*) values, while the x-axis represents the study period, with day 0 marking the day when the last fish was introduced to the tanks, i.e. days post-introduction (dpi). *Cq* values of 40 indicate negative results. ns: *P* > 0.05, *: *P* ≤ 0.05, **: *P* ≤ 0.01, ***: *P* ≤ 0.001, ****: *P* ≤ 0.0001.

The Atlantic salmon housekeeping gene *EF1α* was consistently detected in all swabs and water samples, but not in the aerosol samples (data not shown). Hence, considering the generally high bacterial and fungal load in RAS, the 16S rRNA gene and ITS of *S. parasitica* were used as control genes to validate the aerosol samples. Both the 16S rRNA gene and the ITS of *S. parasitica* were consistently detected at low *Cq* values in biofilter aerosol samples compared to degasser samples only from hall B in Smolt farm 1 (Fig. 3A–D). Significant differences were seen between the Coriolis+ and Coriolis Compact for both control genes from biofilter aerosol samples (Fig. 3A and B). Only the 16S rRNA gene was consistently detected in the local degasser samples, but no significant difference was seen between the two samplers (Statistical analysis for ITS *S. parasitica* not performed due to inconsistent detections at LOD) (Fig. 3C and D). Throughout the study period, consistent detections of all five pathogens were also recorded in the biofilter aerosol samples compared to sporadic detections in the degasser samples (data not shown). Therefore, only biofilter aerosol sample results are presented henceforth (degasser aerosol sample data omitted, except for control genes). All control samples tested negative for all pathogens and housekeeping genes in the study.

### No pathogens were detected in hall A

All baseline gill swab samples collected in hall A tested negative for SGPV, ISAV, and *F. psychrophilum*. No baseline kidney samples were collected; therefore, baseline testing for IPNV and PRV-1 in fish swabs could not be performed. Primary and anaesthetic tank water samples also tested negative for all five pathogens. Furthermore, no pathogens were detected in the baseline aerosol samples (data not shown).

#### The pathogen load in the aerosol reflects the prevalence of gill pathogens detected using gill swabs and water samples

**Fig. 4:**
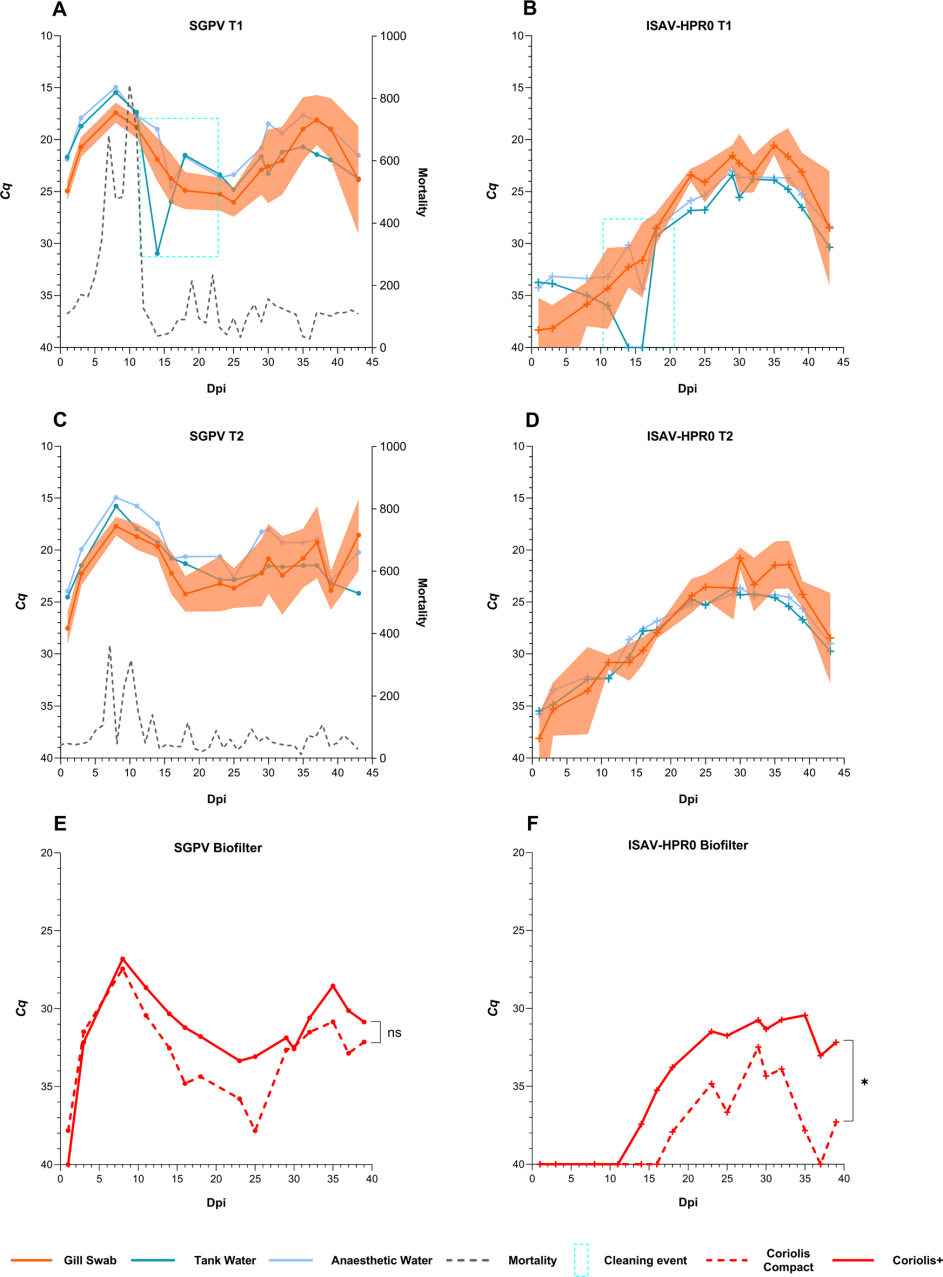
Pathogen dynamics of SGPV and ISAV-HPR0 in gill swabs with concurrent levels in water and aerosol samples. The area surrounding the curves represents the standard deviation. The black curves in the graph indicate daily mortality, with peak mortality observed following the initial peak infection. The y-axis displays the inverted mean cycle quantification (*Cq*) values, while the x-axis represents the study period, with day 0 marking the day when the last fish was introduced to the tanks, i.e. days post-introduction (dpi). The blue rectangle marks the partial cleaning event on the fifth sampling day (14 dpi). *Cq* values of 40 indicate negative results. ns: *P* > 0.05, *: *P* ≤ 0.05, **: *P* ≤ 0.01, ***: *P* ≤ 0.001, ****: *P* ≤ 0.0001.

In hall B, the infection began with classical clinical signs of SGPV, and pale gills were observed during necropsy^38,45^. In both tanks, a similar level of SGPV load, peaking at 7 dpi, was seen, with significantly higher mortality in T1 compared to T2 (Fig. 4A and C). Following the SGPV peak, a transient ISAV infection was observed (Fig. 4B and D) in both tanks. Sequencing confirmed that the ISAV detected was the non-virulent ISAV-HPR0^36^. In T1, a secondary SGPV peak was observed at 35 dpi, concurrent with the peak of ISAV-HPR0 infection. In T2, a more diffuse secondary SGPV peak coincided with the ISAV-HPR0 peak. During the secondary SGPV peak, the on-site veterinarian confirmed that the fish exhibited clinical challenges unrelated to viral infection (data not shown). The infection dynamics of SGPV and ISAV-HPR0 in the gill swab samples were reflected in both the primary and anaesthetic tank water, with the anaesthetic water producing curves more similar to the gill swabs than the primary water (Fig. 4A–D). The anaesthetic tank water appeared to mitigate the effects of partial cleaning in Tank 1. The reduced pathogen levels observed in the primary tank water for both SGPV and ISAV-HPR0 at 14 dpi were not observed in the anaesthetic water samples (highlighted by the blue rectangle; Fig. 4A and B). Correlation analysis showed a stronger positive correlation between anaesthetic tank water (AT) and gill swab samples than between primary tank water (PT) and gill swab samples for SGPV [*r*_*s*_*/P*—PT: 0.63/<0.05(T1), 0.50/<0.05(T2) vs AT: 0.88/<0.0001 (T1), 0.79/<0.001 (T2)] and ISAV-HPR0 [*r*_*s*_*/P*—PT: 0.86/<0.0001 (T1), 0.89/<0.0001 (T2) vs AT: 0.90/<0.0001 (T1), 0.94/<0.0001 (T2)]. Corresponding to the SGPV and ISAV-HPR0 peaks observed in gill swab and water samples, aerosol samples from the biofilter room also displayed similar peak curves, but at a much lower magnitude (~ 5–10 *Cq* value corresponding to a 100–1000 fold lower level of SGPV and ISAV-HPR0 in the aerosols) (Fig. 4E and F). Overall, Coriolis+ performed marginally better than the Coriolis Compact, with lower *Cq* values of ~ 2–3 (Fig. 4E and F). Sporadic detections of SGPV and ISAV-HPR0 were also observed in the aerosol samples from local degassers of both tanks (data not shown). A strong positive correlation was also observed between water (mean *Cq* of T1 and T2) and biofilter aerosol samples for both samplers for SGPV [*r*_*s*_*/P*: 0.77/≤0.001 (Coriolis Compact), 0.53/<0.05 (Coriolis+)] and ISAV-HPR0 [*r*_*s*_*/P*: 0.82/<0.001 (Coriolis Compact), 0.90/<0.000 (Coriolis+)].

#### Internal pathogens were poorly represented in aerosol samples

**Fig. 5:**
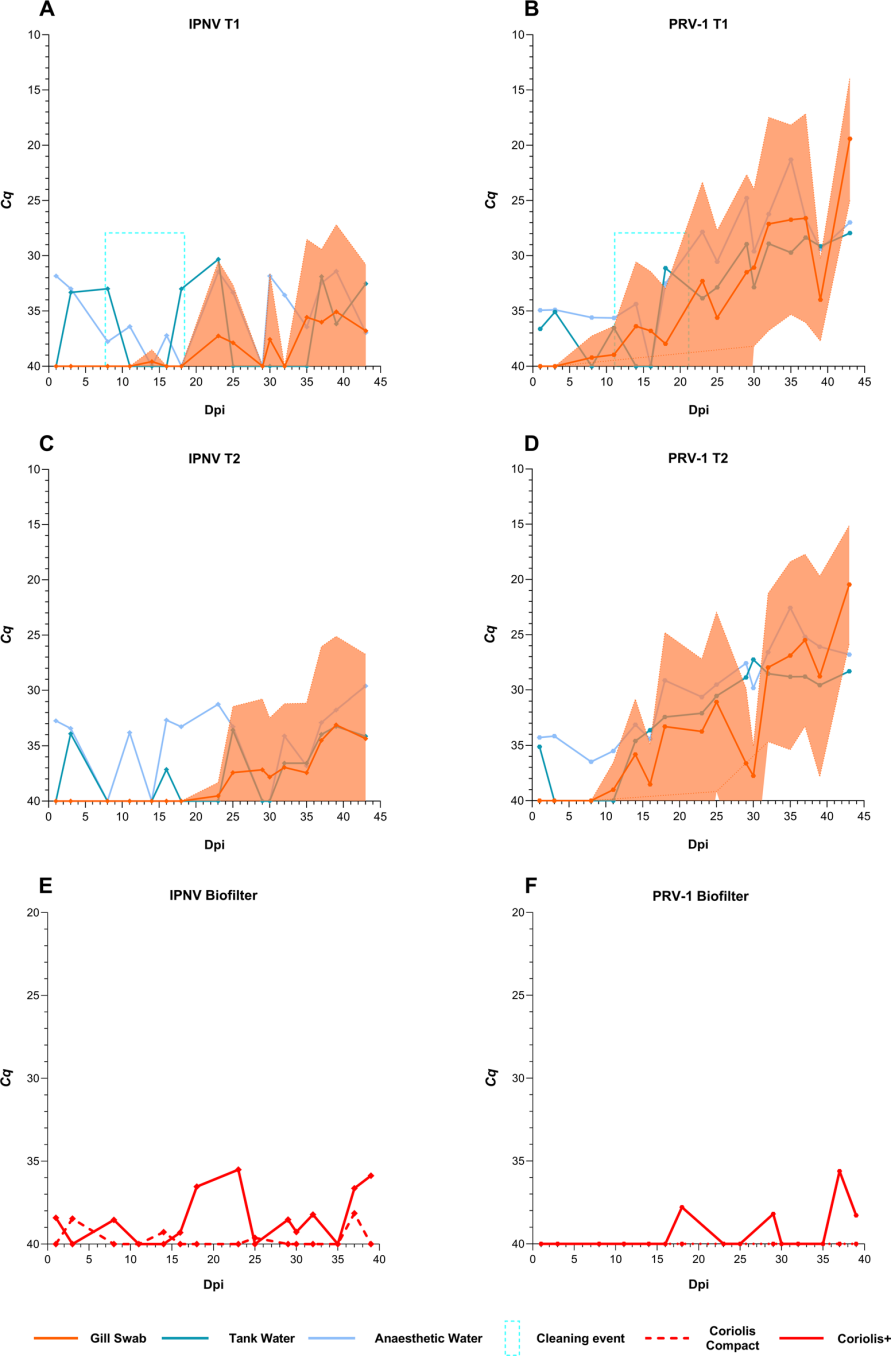
Pathogen dynamics of IPNV and PRV-1 in kidney swabs with concurrent levels in water and aerosol samples. The area surrounding the curves represents the standard deviation. The y-axis displays the inverted mean cycle quantification (*Cq*) values, while the x-axis represents the study period, with day 0 marking the day when the last fish was introduced to the tanks, i.e. days post-introduction (dpi). The blue rectangle marks the partial cleaning event on the fifth sampling day (14 dpi). *Cq* values of 40 indicate negative results.

IPNV was intermittently detected in kidney swab samples from both tanks in hall B (Fig. 5A and C). Concurrently, PRV-1 infection exhibited a progressive trend in both tanks, peaking on the final sampling day (Fig. 5B and D). The peak of PRV-1 infection was seen post-peak ISAV-HPR0 and secondary SGPV peak. No significant mortality was observed during the PRV-1 infection peak. Unlike SGPV and ISAV-HPR0, the effect of partial cleaning of tank T1 was not observed for IPNV and PRV-1 at 14 dpi. IPNV and PRV-1 also showed higher detection rates and lower *Cq* values in anaesthetic tank water compared to primary tank water. Correlation analyses indicated a strong positive correlation between kidney swab and primary tank water for IPNV only in T2, with no correlation observed between kidney swab and anaesthetic water in either tank [*r*_*s*_*/P*—PT: 0.30/>0.05 (T1), 0.6/<0.05 (T2) vs *r*_*s*_*/P*—AT: 0.45/>0.05 (T1), 0.26/>0.05 (T2)]. In contrast, PRV-1 showed a strong positive correlation between kidney swab and primary water, with an even stronger correlation with anaesthetic water [*r*_*s*_*/P*—PT: 0.80/<0.001 (T1), 0.76/≤0.001 (T2) vs *r*_*s*_*/P*—AT: 0.85/<0.0001 (T1), T2: 0.89/<0.0001 (T2)]. Both viruses were poorly represented in aerosol samples, with detections at the RT-qPCR limit of detection (LOD) at all positions; however, IPNV had slightly higher detection rates than PRV-1. Coriolis+ outperformed Coriolis Compact in capturing both the internal pathogens (Fig. 5E and F**)**. No correlation analysis was performed for water and aerosol samples.

#### Detection of an opportunistic bacterial pathogen in aerosol samples

**Fig. 6:**
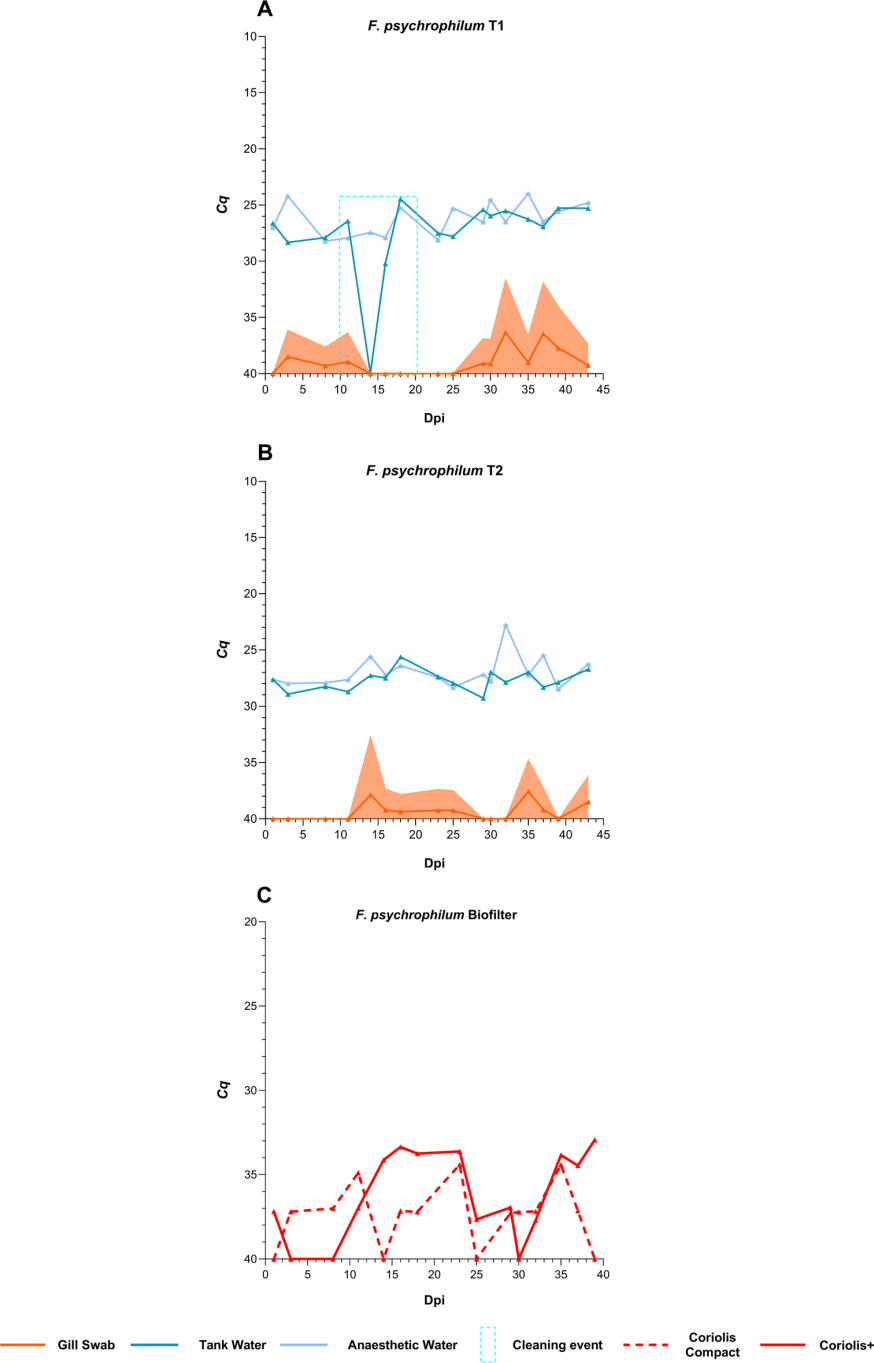
Pathogen dynamics of *F. psychrophilum* in gill swabs with concurrent levels in water and aerosol samples. The area surrounding the curves represents the standard deviation. The y-axis displays the inverted mean cycle quantification (*Cq*) values, while the x-axis represents the study period, with day 0 marking the day when the last fish was introduced to the tanks, i.e. days post-introduction (dpi). The blue rectangle marks the partial cleaning event on the fifth sampling day (14 dpi). *Cq* values of 40 indicate negative results.

*F. psychrophilum* was detected sporadically at the LOD in gill swab samples with no evident clinical signs. In the water samples, *F. psychrophilum* levels remained consistently higher (lower *Cq* values) in both primary and anaesthetic water samples in both tanks (Fig. 6A and B). Partial cleaning of T1 had a notable impact, with bacterial levels drastically falling in the primary tank water sample at 14 dpi (Fig. 6A). The bacterial levels remained unaffected in the anaesthetic water samples. No correlation analysis was performed for gill swabs and the two water sample types. In the aerosol samples, the bacterium was mainly detected in the biofilter aerosol samples (Fig. 6C), with *Cq* values fluctuating between *Cq* 35 and LOD. Both Coriolis+ and Coriolis Compact performed similarly, with no significant and consistent detection pattern. There was no correlation between the water and aerosol samples from either sampler.

### Isolation of viable bacterial species from aerosol samples

To determine if the aerosol samplers captured viable *Flavobacterium psychrophilum*, direct culture of aerosol samples from Smolt farm 1 was performed on Cytophaga agar (CA). Direct culture produced fewer than 5 CFU per CA plate, mainly from the biofilter aerosol samples. Most bacterial growth was observed in samples collected using the Coriolis Compact. Ten distinct colonies, distinguished by their morphology, were subcultured on CA and Sheep Blood agar (BA) to obtain pure cultures and prevent fungal contamination. No *F. psychrophilum* was identified based on 16S rRNA gene sequence identity. RAS-relevant environmental bacteria (colony count, percentage similarity based on BLAST, and accession number are indicated inside parentheses), like *Acidovorax sp*. (1; 99.39%; PV876915), *Hymenobacter setariae* (1; 97.69%; PV876906), *Microbacterium sp*. (4; 99.79–99.86%; PV876910, PV876912, PV876913, PV876914), *Rhodococcus sp. and R. qingshengii* (2; 99.12–100%; PV876907, PV876909), and *Staphylococcus equorum* (2; 99.86–100%; PV876908, PV876911) were isolated from the aerosol samples.

### Isolation of viable aerosolised infectious pancreatic necrosis virus (IPNV)

To determine if the aerosol samplers isolated viable IPNV, IPNV-positive aerosol samples collected from Smolt farm 1 were inoculated on BF-2 and EPC cell lines. After two weeks of incubation, non-characteristic/unknown cytopathic effects (CPE), unrelated to IPNV, were observed. Due to the nonspecific destruction of cell monolayers at the end of the two weeks, the samples were not passaged onto a fresh cell monolayer. All cell supernatants tested negative with RT-qPCR for IPNV, and other viral pathogens routinely tested at FFVA (data not shown). Therefore, targeted sampling was performed in Smolt farm 2 following a clinical outbreak of IPNV. IPNV was detected in water samples collected after the peak of the outbreak, with two of the triplicate water samples testing positive for IPNV (Avg. *Cq* 33). In Coriolis Compact samples, IPNV was detected in two replicates (Avg. *Cq* 36), while only one sample from Coriolis+ samples tested positive at *Cq* 34 (post concentration). Expedited processing of aerosol samples from both samplers and maintaining a strict cold chain had a notable impact on the isolation of viable IPNV from aerosols. Characteristic CPE for IPNV was observed after 72 h of incubation in the BF-2 cell line, while changes in the EPC cell line were observed after seven days in samples collected with the Coriolis Compact only. After seven days, the entire cell culture plates were passaged again. In the second passage, CPE was detected within 48 h in the BF-2 cell line and after 72 h in the EPC cell line. Wells showing CPE were confirmed positive for IPNV infection using RT-qPCR (data not shown).

### Two distinct infectious pancreatic necrosis virus (IPNV) variants isolated from smolt farm 2

Two distinct variants of IPNV, each with different SNP profiles, were identified in fish samples from Smolt farm 2 (Table 2). Classification of variants was based on the IPNV virulence determinants, mainly at amino acid positions aa_217_, aa_221_ and aa_247_, with both variants displaying additional amino changes not typical of classical low-virulent IPNV^46–49^. Additionally, the variants in this study caused outbreaks in the fish at Smolt farm 2, demonstrating the development of new variants capable of causing infectious pancreatic necrosis (IPN) in genetically IPNV-resistant (QTL-IPN) Atlantic salmon. The amino acid profile of IPNV from fish and cell culture isolates, highlighting the two variants, is detailed in Table 2. The cell culture isolates matched one of the variants found in the fish samples. In the first cell culture passage, we report an additional SNP mutation detected in both BF-2 and EPC cell lines, leading to an amino acid change at aa_141_ from leucine → valine (highlighted in red), which reverted from valine → leucine (matching fish tissue samples) in the second passage.

**Table 2:**
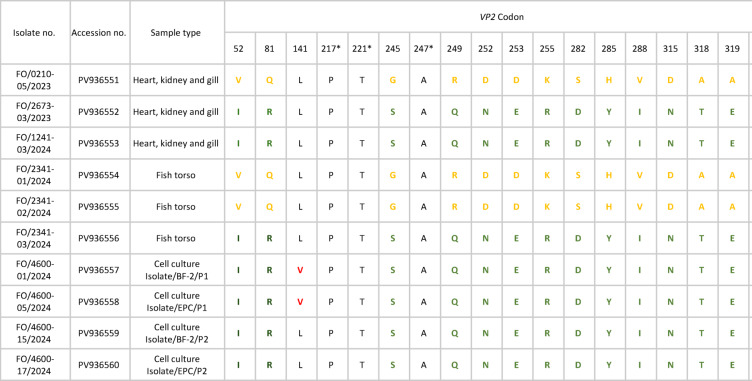
Amino acid configuration at their respective positions with substitutions at the *VP2* codon in fish samples and cell culture isolates collected from smolt farm 2.

The amino acid change in the variants in fish isolates is highlighted in different colours (green and yellow). The variants were determined as low-virulent based on the virulence determinants (*) previously described^46–49^. Cell culture isolates displayed an additional amino acid variation at aa_141_ in the first passage (highlighted in red), with a reversal to the amino acid seen in the fish isolates in the second passage. BF-2 and EPC are the two cell lines, with P1 and P2 indicating the first and second passages, respectively.

## Discussion

To our knowledge, this is the first study demonstrating probable aerosol-mediated transmission of the fish viruses SGPV, ISAV-HPR0, and IPNV in RAS. We provide the first field-based evidence of viable IPNV isolated from aerosols at a commercial Atlantic salmon RAS farm, along with insights into infection dynamics, the predictive value of water enhanced using anaesthetic water, and the presence of multiple Atlantic salmon pathogens in aerosol samples. Furthermore, we show that pathogen load in aerosols is directly influenced by their levels in the RAS water.

### Infection dynamics of fish pathogens SGPV and ISAV-HPR0 are reflected in the aerosol samples

In our previous study, we have elucidated the infection dynamics of SGPV, ISAV-HPR0, IPNV, PRV-1, and *F. psychrophilum* in two RAS systems at Smolt farm 1, detailing the complex infection patterns within RAS systems, and demonstrated that water samples offer a non-lethal alternative to fish sampling^28^. In this study, we employed a similar sampling strategy to replicate these findings in another fish group at the same Smolt farm 1 and to elucidate the temporal and spatial distribution of pathogens in aerosol samples. We were successful in replicating the complex infection dynamics, in turn reflected by the water and aerosol samples. The infection in the RAS began with a clinical outbreak of SGPV as previously described^28^. The onset of infection/disease occurred approximately two weeks after the fish were relocated from a naïve system to a system containing the studied pathogens and could be attributed to the stress^45^ associated with the moving process, as previously reported^28^. The SGPV infection may also have acted as a trigger for other potential pathogens, as a transient ISAV-HPR0 infection was detected in the gills, post-SGPV infection. Severe damage to the gill epithelium and immune suppression may have facilitated the secondary transient ISAV-HPR0 infection^50,51^. Both SGPV and ISAV-HPR0, which were previously reported in this RAS system^28^, may have persisted due to incomplete disinfection.

The infection dynamics of the gill pathogens SGPV and ISAV-HPR0 were also closely reflected in the water samples, as previously described^28^, and in turn also reflected in the aerosol samples. Both temporally and spatially, the highest detection rates in aerosols from both samplers were for SGPV, followed by ISAV-HPR0, particularly in the biofilter samples, with sporadic detection in the local degasser samples. A study by Affek et al.^52^ concluded that biofilters could be a source of pathogenic microorganisms, with the type of biofilter and the number of microbes in the bed significantly affecting bioaerosol emissions in an industrial plant. SGPV and ISAV-HPR0 were captured more effectively compared to other pathogens in the study, possibly due to the higher prevalence of these pathogens in the water samples as a result of epithelial shedding^38,50,53^. Viral load of SGPV and ISAV-HPR0 in aerosol samples was 100-1000-fold lower (5–10 *Cq* higher) than in water samples, potentially due to aerosol formation characteristics, relative humidity, temperature, and other environmental factors^54^, leading to higher dilution of pathogen levels in air.

Elucidating the infection dynamics of ISAV-HPR0 has been challenging, particularly due to the non-clinical transient nature of the infection and its ability to evade detection^5,36,55^. In our study, we were able to elucidate the infection dynamics of ISAV-HPR0 while revealing that ISAV-HPR0 infection dynamics in gills were reflected in aerosol samples from the biofilter. The detection of ISAV-HPR0 in aerosol samples is particularly important for RAS, as ISAV-HPR0, the precursor to virulent, disease-causing ISAV HPR-deleted variants^56^, can persist as “house strains” in RAS systems^5^ and may spread via aerosols within the RAS farm, much like avian influenza viruses^57^. While ISAV-HPR0 spreads primarily through horizontal transmission via water and infected fish between smolt farms and sea sites, with no evidence of vertical transmission^5^, aerosols may also serve as a potential horizontal transmission route. Christiansen et al.^5^ have hypothesised the introduction of ISAV-HPR0 variants into smolt farms from the marine environment, likely through HPR0-contaminated sea spray. Our findings support this hypothesis by demonstrating that ISAV-HPR0 is readily detected in aerosol samples originating from RAS degassers, simulating sea spray production. While ISAV-HPR0 has the potential to evolve into virulent forms^5,56^, the risk of emergence of ISA outbreaks is low and influenced by management practices, as well as environmental and molecular factors^36,56,58,59^. Nonetheless, aerosolisation presents a biosecurity risk for spreading ISAV-HPR0.

Following SGPV and concurrent ISAV-HPR0 infection, we observed a progressive PRV-1 infection alongside sporadic low levels of IPNV. Previously, we reported co-infection of SGPV with ISAV-HPR0, IPNV, and sporadic PRV-1 in the same RAS^28^. This study revealed a reversed infection pattern with inconsistent pathological findings, where PRV-1 predominated and IPNV occurred sporadically, likely due to subclinical infections in a subpopulation of fish. Nonetheless, like SGPV and ISAV-HPR0, both IPNV and PRV-1 seem to have persisted in the RAS. In contrast to SGPV and ISAV-HPR0, both IPNV and PRV-1 were poorly represented in the aerosol samples; however, PRV-1 showed lower Cq values and higher detection rates in water samples than IPNV. The underrepresentation of IPNV and PRV-1 in aerosol samples warrants further investigation, and no definitive remarks on their dynamics in aerosols can be made at this stage. *Flavobacterium psychrophilum*, an opportunist, was consistently detected only in water samples with sporadic detections and no evident infection in the fish. Research suggests that *F. psychrophilum* can remain undetected until fish become susceptible^60^ and can persist in fish farms^61^, which aligns with our current and previous findings^28^. The bacterium was detected at higher *Cq* values than water samples, close to LOD, with no consistent pattern in the gill swabs and aerosol samples. The bacterial levels were also notably higher in aerosol than in gill swab samples, reinforcing evidence that pathogen concentration in water influences aerosol composition. Overall, this study reveals the complexity of infections in RAS, demonstrating that water recirculation with incomplete disinfection heightens infection risk while emphasising the critical need for strong biosecurity measures, since waterborne fish pathogens can readily become airborne.

### Viable IPNV successfully isolated from Coriolis Compact aerosol samples

Of the four viral pathogens investigated in the current study, only IPNV can be propagated in a cell culture assay, hence serving as a “proxy” in determining the viability of viral pathogens in aerosol samples collected with the Coriolis+ and Coriolis Compact. We were not able to isolate viable IPNV from Smolt farm 1 with the two aerosol samplers, most likely due to a suboptimal sampling strategy. We used a higher concentration of surfactant (Ecosurf EH-9, Triton X-100 replacement; 0.1%) in the sampling liquid, which is known to cause viral inactivation^62^, compared to the lower concentrations of surfactants (Triton X-100; 0.01% − 0.05%) used in previous studies^63,64^. The aerosol samples were also stored long-term (up to 6 months) at − 70 °C before processing and could have impacted viral viability^65^. With a robust, expedited and optimised targeted sampling strategy at Smolt farm 2, using plain DPBS without surfactant as collection/reconstitution liquid and immediate cell culture propagation, IPNV was successfully isolated.

Routine sampling at Smolt farm 2 revealed two variants of IPNV circulating in the RAS, causing a clinical outbreak. Genotyping of IPNV isolated from cell culture revealed that the airborne IPNV variant matched one of the variants found in fish samples during the outbreak at Smolt farm 2. Analysis of the partial VP2 gene of IPNV from cell culture and previous fish samples collected from Smolt farm 2 revealed proline, threonine, and alanine at amino acid positions aa_217,_ aa_221_, and aa_247,_ respectively, mutations linked to reduced replication and evolutionary rates and hence designated as low virulent variants^46,47^. However, because these variants were able to cause outbreaks in QTL-IPN resistant fish and harbour additional amino acid changes, they represent new non-classical low-virulent IPNV capable of causing mortality and outbreaks in genetically resistant Atlantic salmon, as previously reported by Hillestad et al. ^48^ and Godoy et al. ^49^. The IPNV isolated from the two cell lines displayed an additional SNP resulting in an amino acid change at aa_141_, from leucine to valine, in both BF-2 and EPC during the first passage and reverted to the amino acid configuration, leucine_141_, resembling fish isolates in the second passage. This amino acid change at aa_141_ has not been previously documented and is not considered a virulence determinant, as described by Dopazo^47^, but it may indicate the virus’s acclimatisation from one cell type to another. Santi et al.^40^ reported a similar phenomenon, where they observed a rapid amino acid substitution from Alanine to Threonine at position aa_221_ after multiple cell culture passages, indicating potential attenuation and reduced virulence. In contrast, Gadan et al.^66^ showed reversion to virulence from attenuation; however, we did not observe these changes in virulence-associated amino acids in our cell culture isolates. A change in the infected host cell type may have challenged IPNV in the aerosol samples introduced into the cell monolayer during the first passage, resulting in the amino acid change at aa_141_ as a form of adaptation. Previously, only non-viral fish pathogens have been isolated from aerosol samples^17–21^, marking the current findings the first field-based evidence of isolation of a viable fish viral pathogen from aerosol samples from Atlantic salmon RAS.

### RAS-relevant environmental bacteria isolated from aerosol samples

Alongside isolating viable IPNV, our study attempted but failed to isolate viable *F. psychrophilum* from aerosol samples from RAS. The unsuccessful isolation of *F. psychrophilum* is possibly due to the absence of apparent infection in fish and high *Cq* values in the aerosol samples, coupled with long-term storage at − 70 °C^67^. In contrast, we isolated a few bacterial colonies, which were identified as *Acidovorax spp.*, *Hymenobacter setariae*, *Microbacterium spp.*,* Rhodococcus spp.*,* Rhodococcus qingshengii*, and *Staphylococcus equorum*. The number of isolated colonies was low, possibly because Cytophaga agar is recommended specifically for *Cytophaga spp.* and *Flavobacterium spp.* Among the environmental bacteria identified in our study, *Acidovorax spp*. can be found in the biofilter as part of the nitrate cycling communities^68^ as well as packaged Atlantic salmon fillet^69^. *Hymenobacter spp.* have been isolated from diverse environmental habitats, including air samples^70^. *Microbacterium spp.* are found in marine environments^71^ and have been isolated from the gut of Atlantic salmon^72,73^, raw frozen cultured seafood^74^ and the environment of Atlantic salmon processing factories^75^. *Rhodococcus spp*. are known to be associated with freshwater and marine recirculating aquaculture systems^76^, with *Rhodococcus qingshengii* found on pseudo-membranes covering the internal organs of Atlantic salmon and identified as a potential opportunistic pathogen^77^. *Staphylococcus equorum* has been identified in traditional air-dried Faroese sheep meat^78^. The bacterium has also been isolated from Atlantic salmon gut^72,73^, and is likely widespread in the environment. In summary, isolating bacteria from aerosol samples sheds light on the possible role of aerosols in transmitting fish bacterial pathogens, as demonstrated by Gołaś et al.^20^, who identified the potentially human-pathogenic *Aeromonas hydrophila* in aerosols produced by water droplets from RAS systems. Overall, even when the pathogen of interest was not isolated, the successful detection of diverse environmental bacteria from RAS aerosols demonstrates their potential as a valuable tool for monitoring RAS health. Further research on RAS aerosols could offer practical insights into hygiene and aerosol microbial community dynamics.

### Anaesthetic water is a potentiated non-lethal sample that improves pathogen detection

We have previously demonstrated that water samples provide a reliable and non-lethal method for detecting and monitoring fish pathogens, SGPV and ISAV-HPR0 in Atlantic salmon RAS^28^. In the present study, we expand on these findings by using anaesthetic water as an enhanced sampling medium. SGPV, ISAV-HPR0, and *F. psychrophilum* consistently showed higher detection rates in anaesthetic water compared to primary tank water, with no clear impact of the cleaning event observed in this sample type. For the gill pathogens SGPV and ISAV-HPR0, detection in anaesthetic water strongly mirrored infection dynamics with strong correlation to fish samples, likely due to rapid epithelial shedding^45,53,79^, in line with previous findings^28^. *F. psychrophilum* was also detected at higher levels in water than in fish, with slightly lower *Cq* values in anaesthetic water, suggesting increased shedding, potentially from moribund fish^80^. However, this signal reflects the presence of the bacterium in the RAS rather than the individual infection status in fish. Detection rates for IPNV and PRV-1 were higher in anaesthetic water than in primary tank water. While PRV-1 levels in kidney swabs correlated more strongly with those in anaesthetic water compared to IPNV, no consistent correlation with fish viral load was found for either virus, as the study did not capture the full infection course for either virus. According to Polinski et al.^81^, water samples were reliable only when fish samples were PRV-1 positive, and offered limited information on the virus’s prevalence and transmission. Because of the subclinical nature of infections in our study, no firm conclusions could be drawn about their shedding patterns or the diagnostic value of water samples for these internal pathogens. Nevertheless, these findings suggest that anaesthetic water can enhance non-lethal detection of certain pathogens like SGPV and ISAV-HPR0 in RAS, and that the handling process may facilitate pathogen release into the surrounding water. The smaller, defined volume of anaesthetic water likely contributes to the up-concentration of target material, improving detection sensitivity. Thus, anaesthetic water may serve as a useful indicator of system-level pathogen presence, but it cannot be used as a standalone diagnostic tool.

### Coriolis+ offers higher sensitivity

In this study, we compared the performance/feasibility and sensitivity of two air samplers, the Coriolis+ and Coriolis Compact, in detecting specific fish pathogens in RAS. In the initial validation using the 16S rRNA gene and ITS of *S. parasitica*, Coriolis+ performed significantly better than Coriolis Compact. Coriolis+ also demonstrated a higher sensitivity in detecting all pathogens in the current study, reflecting the infection dynamics of SGPV and ISAV-HPR0 more accurately than the Coriolis Compact, even with modest sampling efficiency^63,82^. The increased sensitivity could be a result of Coriolis+ sample up concentration using centrifugal concentrators/ultrafiltration units. A recent study by Hallak et al.^83^ involving Coriolis+ has suggested methodological optimisation to increase its efficiency and reliability in bioaerosol research. Low representation of internal pathogens in aerosol samples, especially from Coriolis Compact, likely resulted from subclinical infections affecting only a subset of fish, limiting their levels in aerosols. This finding highlights the importance of an active infection and viral shedding into surrounding water in contributing to the pathogen levels in the aerosols, as witnessed from the results of two distinct groups (gill and internal) of pathogens in our study. *F. psychrophilum* was also poorly represented in the two aerosol samplers, as no apparent bacterial infection was observed, further highlighting the role of active infection in evaluating the sampler performance and sensitivity. Despite the slightly lower sensitivity of Coriolis Compact in pathogen detection, viable IPNV was successfully isolated from the Coriolis Compact aerosol samples containing low viral load and not from the Coriolis+, post-targeted sampling. The disparity in collecting viable viral particles may be attributed to the difference in the aerosol collection principles of the two samplers (Bertin Technologies SAS, France). Nonetheless, the Coriolis Compact also offers advantages during indoor operation due to its compact design and ease of use, and can function autonomously for up to 8 h, compared to just 1 h for the Coriolis+ (without the long-term monitoring platform) (Bertin Technologies SAS, France). Coriolis Compact also eliminates the collection fluid requirement that can lead to fluid and biological material losses associated with fluid-based impingement sampling with Coriolis+^83^. The ultimate choice of sampler depends on the nature of the study, suitability of its application in the field and final research goal, as no single sampler has universal application^82^. Our results provide field-based insights into the performance and sensitivity of samplers for detecting fish pathogens, highlighting how performance varies with sampling conditions^82^, and the significance of active infections in evaluating sampler efficiency.

## Conclusion

Studies have already established the role of the sea surface microlayer in harbouring and transmitting viruses and bacteria over long distances^22,23^. Our findings build on the sea-spray mediated transmission hypothesis proposed by Christiansen et al.^5^ and indicate that RAS degassers may promote the aerosolisation and spread of fish pathogens in a manner similar to sea spray. When considering the overall scale of a RAS importing huge amounts of air, the presence of multiple systems, and the continuous production nature, our results indicate a constant generation of pathogen-laden aerosols during outbreaks. Depending on the proximity of the RAS systems as well as RAS farms to each other and the marine grow-out cages/sites, the potential for aerosol-mediated transmission remains high, as the inlet and outlet air are not decontaminated. Furthermore, the isolation of viable IPNV as a “proxy” for non-vertically transmitted ISAV-HPR0 underscores the biosecurity risk, since ISAV-HPR0 is frequently detected at smolt farms and marine sites without apparent transmission routes. With some degree of caution, based on the detection of viral RNA and the successful isolation of IPNV from aerosol samples, it can be inferred that aerosols may have the potential to transmit fish viral pathogens. Finally, building on our previous research^28^, while anaesthetic water sampling is more effective than standard water sampling for pathogen detection, its utility is limited to assessing system-wide pathogen prevalence and does not extend to disease diagnosis.

## Limitations and future scope

The current study was conducted at a commercial RAS facility. Fish samples were collected during routine culture operations, with a focus on freshly dead and moribund fish (semi-targeted sampling). Due to the nature of the facility, there was limited control over environmental parameters. The results also indicate that management practices at the facility influenced certain aspects of the findings. For aerosol sampling, only one sample per location was collected, providing a snapshot of the pathogens present in the aerosol at the time of sampling. The samplers used were commercially validated, and their efficiency was assessed based on their performance and pathogen detection levels/rates due to the observational nature of the study. The performance was validated based on the detection rates and levels of the 16S rRNA gene and ITS of *S. parasitica*. These limitations could be addressed in an infection trial under controlled experimental settings, while simultaneously measuring the efficiency of the aerosol samplers for fish pathogens as well as the impact of RAS management practices on the collection process. While the current study demonstrates the presence of viable viruses in aerosol with high potential for transmission, a phylogenetic assessment of samples from multiple RAS and sea sites would provide insights into the epidemiology.

## Data Availability

The sequences generated in this study have been deposited in the GenBank database under accession numbers PV876906-PV876915 and PV936551-PV936560. All other data supporting the conclusions of this article are included within the article as main texts, figures and tables.
